# Disease-specific haptoglobin-β chain N-glycosylation as biomarker to differentiate non-small cell lung cancer from benign lung diseases

**DOI:** 10.7150/jca.32690

**Published:** 2019-09-07

**Authors:** Tianjing Chen, Chengyan He, Mo Zhang, Xiaoou Li, Xiaofeng Liu, Yujie Liu, Dan Zhang, Zhili Li

**Affiliations:** 1 Department of Biophysics and Structural Biology, Institute of Basic Medical Sciences, Chinese Academy of Medical Sciences & School of Basic Medicine, Peking Union Medical College, Beijing, PR China; 2 Clinical Lab Diagnosis, China-Japan Union Hospital, Jilin University, Changchun, PR China; 3 Department of Laboratory, Tumor Hospital of Jilin Province, Changchun, PR China

**Keywords:** disease-specific haptoglobin β chain, glycosylation, lung diseases, pathological state, matrix assisted laser desorption/ionization-mass spectrometry.

## Abstract

**Background**: The association of pathological states with N-glycosylation of haptoglobin-β has attracted increasing attention.

**Materials & Methods**: In the present study, disease-specific haptoglobin-β (DSHp-β) was separated from serum immunoinflammation-related protein complexes (IIRPCs) of 600 participants including 300 patients with benign lung diseases (BLDs) and 300 patients with non-small cell lung cancer (NSCLC). The enriched glycopeptides of the tryptic digests of the DSHp-β were analyzed using matrix assisted laser desorption/ionization-Fourier transform ion cyclotron resonance mass spectrometry (MALDI-FTICR MS).

**Results**: 20 of glycopeptides were detected for each sample. The statistical analysis has indicated that significant changes in the sialylation of DSHp-β between BLDs and NSCLC patients were observed. The age- and sex-matched participants were randomly clarified into the training set and the validation set. Receiver operating characteristic (ROC) analysis has revealed that the level ratio of glycopeptides (G2G3/G2G3S4) at the sites of Asn207/211 has potential capability to distinguish BLDs from NSCLC, with the sensitivity of 74.4%, the specificity of 82.8%, and the area under curve (AUC) of 0.805.

**Conclusion**: The glycosylation of DSHp-β can distinguish NSCLC from BLDs with high diagnostic accuracy compared with current clinical available serum markers.

## Introduction

Lung cancer is the leading cause of cancer-death for men and the second cause of cancer-death for women worldwide 1. In China, the incidence of lung cancer also ranks as the first for males and the second for females, making it to be one of the major public health problems 2. Non-small cell lung cancer (NSCLC) includes three major histologic subtypes: adenocarcinoma, squamous cell carcinoma, and large cell carcinoma 3, which accounts for approximately 85% of lung cancer. Due to the late diagnosis of NSCLC, it has a poor prognosis and the five-year survival rate is only 15% 4. Recent improvements in radiographic imaging techniques on pulmonary nodules can distinguish benign from malignant nodules with lung cancer screening and minimally invasive surgical approaches 5, 6. Although the clinical features and imageological examinations such as computed tomography, positron emission tomography, and endobronchial ultrasound are helpful in differentiating between lung cancer and benign lung diseases (BLDs) 7, 8, the final diagnosis still depends on the pathological examination of tissue biopsy. Therefore, the screening effective test which is based on serum biomarkers to distinguish BLDs from lung cancer is of great importance.

Glycans play key roles in both molecular and cellular events of inflammation, the events of which are closely correlated with glycan structure and function 9. Change in sialylation obviously impacts tumor cell interaction with tumor microenvironment and the alteration of glycan structures with terminal sialic acid residue is one hallmark of cancer^10^. Haptoglobin (Hp) is an unique acute phase protein that primarily scavenges haemoglobin, which is released into the circulation by haemolysis and/or normal red blood cell turnover^11^.The aberrant sialylation and fucosylation of Hp-β chain are correlated with cancer^12^, ^13^. Recent studies of total serum haptoglobin fucosylation mainly focused on the comparison between lung cancer and normal control or inflammatory lung diseases 14, 15. Changes in the glycosylation of Hp-β chain with four glycosylation sites (Asn184, Asn207, Asn211, and Asn241) are closely associated with different liver diseases 16. The increased N-glycopeptides of Hp-β in liver diseases were associated with the increased branching with hyperfucosylation and sialylation compared to healthy controls and the significantly increased N-glycopeptides on Asn184 and Asn241 of Hp-β in early hepatocellular carcinoma were observed compared to cirrhosis and normal controls 17. Three glycopeptides (Asn211 and Asn241) of the immunoaffinity-enriched serum Hp-β exhibited high diagnostic accuracy to differentiate healthy controls form gastric cancer using healthy controls (n=15) and gastric cancer (n=10) 18. Sufficient sensitivity approach has allowed the characterization of site-specific glycosylation from trace levels of Hp-β 19.

Previous studies have indicated that serum immunoinflammation-related protein complexes (IIRPCs) are strongly associated with disease states, disease types, and lung cancer progression 20-22. Significant increase in the levels of serum IIRPCs in lung cancer patients was observed compared to BLDs patients 17 and disease specific Hp-β (DSHp-β) chain is one of the major components of serum IIRPCs 22. Change in glycosylation of Hp-β chain may play an important role in occurrence of lung cancer 23. In the present study, a combined strategy of the optimized native polyacrylamide gel electrophoresis (native-PAGE) and sodium dodecylsulfonate-PAGE (SDS-PAGE) was used to separate DSHp-β chain from serum samples, and changes in the glycosylation of DSHp-β between NSCLC and BLDs were detected using matrix-assisted laser desorption ionization-Fourier transform ion cyclotron resonance mass spectrometry (MALDI-FTICR MS). Statistical analysis indicated that level ratio of glycopeptides (G2G3/G2G3S4) at the sites of Asn207/211 has potential capability to differentiate NSCLC from BLDs, with the sensitivity of 74.4% and the specificity of 82.8%.

## Materials and Methods

### Materials

Dithiothreitol (DTT), Iodoacetamide (IAM), 2,5-dihydroxybenzoic acid (DHB), and thyroglobulin were purchased from Sigma-Aldrich (St. Louis, MO, USA). Trypsin (sequencing grade modified) was from Roche Diagnostics GmbH (Mannheim, Germany). Coomassie brilliant blue G250 was acquired from Amresco LLC (Solon, OH, USA). SDS was from USB Corporation (Cleveland, OH, USA). Ammonium bicarbonate was from Sigma-Fluka Corporation (Steinheim, Germany). Acetonitrile (ACN) and trifluoroacetic acid (TFA) of liquid chromatography/mass spectrometry grade were from Fisher Scientific Corporation (Fair Lawn, NJ, USA). Ultrapure water was prepared using a Millipore synergy ultrapure water purification system (Millipore, France). All other reagents are of analytical grade.

### Serum sample collection

In this study, all samples from 600 participants with more than of 12 h fasting were the remaining sera after clinical laboratory examination, and approximately 0.4 mL of serum was collected from each participant. Serum was centrifuged at 3000 g for 10 min at 4°C. The supernatant was harvested followed by dividing into 200 μL aliquots, and immediately stored at -80°Cuntil analysis. Sera of all patients were collected at diagnosis. This study was approved by the Ethics Review Board at the Institute of Basic Medical Sciences, Chinese Academy of Medical Sciences, and informed consent was acquired from each patient. All experiments were performed in accordance with relevant guidelines and regulations.

### Study design

**Figure 1:**
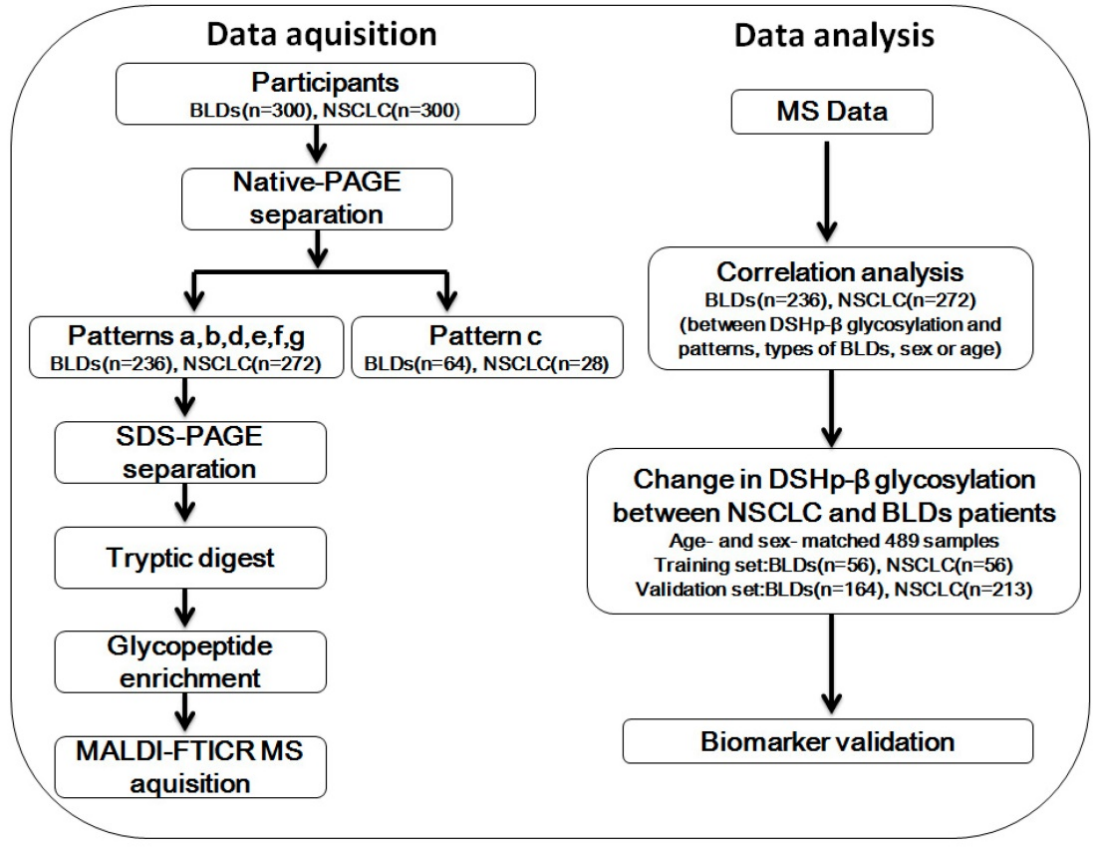
Workflow chart. NSCLC, non-small cell lung cancer; BLDs, benign lung diseases; native-PAGE, native polyacrylamide gel electrophoresis; SDS-PAGE, sodium dodecylsulfate-polyacrylamide gel electrophoresis; MALDI-FTICR MS, matrix-assisted laser desorption/ionization-Fourier transform ion cyclotron resonance mass spectrometer.

In this study, firstly we isolated serum IIRPCs of all participants by native-PAGE and then classified the patterns of the IIRPCs based on their number and location on gels as described previously (**Figure S1A**) 22. For the remaining 508 participants with serum IIRPCs, DSHp-β chain, one component of the IIRPCs, was separated by SDS-PAGE and then digested in gel using trypsin. The detailed characteristics of the 508 participants are listed in **Table 1**. The enriched glycopeptides were detected by MALDI-FTICR MS. Correlation analyses between DSHp-β glycosylation and patterns of the IIRPCs, cancer staging, types of BLDs, sex, or age were performed. Finally, the age-and sex-matched 489 participants were randomly divided into the training set and independent validation set (**Figure 1**).

### Separation of serum IIRPCs

Serum IIRPCs were isolated by native-PAGE as previously described with slight modification 22. Briefly, 10 μL of serum was separated by a combination of separating gel (4% to 10% gradient acrylamide gel) and stacking gel (4%) to obtain serum IIRPCs. Each gel included nine serum samples and one quality control (QC) sample. The QC sample was the mixed sera of 3 patients with lung diseases. Electrophoresis was run at 10 mA per gel for 1.5 h, followed by 25 mA per gel for 3 h. The gels were stained overnight with Coomassie brilliant blue G-250 followed by being destained in water for at least 24 h.

### Isolation of DSHp-β

The gel bands of serum IIRPCs were excised and washed by ultrapure water, and then serum IIRPCs were in-gel reduced with 300 μL of 0.2 M DTT for 45 min at 37°C, followed by alkylation with 300 μL of 0.5 M IAM for other 45 min. After being washed by ultrapure water, the gel bands were allowed for SDS-PAGE separation. Electrophoresis was performed at 60 V for 45 min, followed by 120 V for 1.5 h. Then the gels were stained with Coomassie brilliant blue G-250 (**Figure S2**).

### Tryptic digestion and glycopeptide enrichment

All bands containing DSHp-β from one patient in SDS-PAGE were put together and then cut into pieces. 50% ACN in 25 mM ammonium bicarbonate was used for destaining followed by dehydration in 100% ACN. 10 μL of trypsin (12.5 ng/μL in 25 mM ammonium bicarbonate) was added to gel pieces and incubated at 37°C overnight. The supernatant was collected and concentrated using a SpeedVac concentrator to obtain concentrated tryptic digests. Glycopeptides were enriched from the tryptic digests as described previously 24. Briefly, 2 mg of g-C_3_N_4_ was dispersed in 1 mL of 80% ACN. After being vortexed for 30 s, 60 μL of the dispersion liquid was put into the tryptic digests and then vortexed for other 30 s. The mixture was rotated in the dark for 1 h and then centrifuged at 15000 g for 5 min. The supernatant was removed followed by the addition of 60 μL of 0.025% ammonium hydroxide. After being vortexed for 30 s, the solution was rotated in the dark for 40 min followed by the centrifugation at 15000 g for 5 min. The supernatant containing glycopeptides was collected and concentrated using a SpeedVac concentrator.

### Mass spectrometry analysis

**Figure 2:**
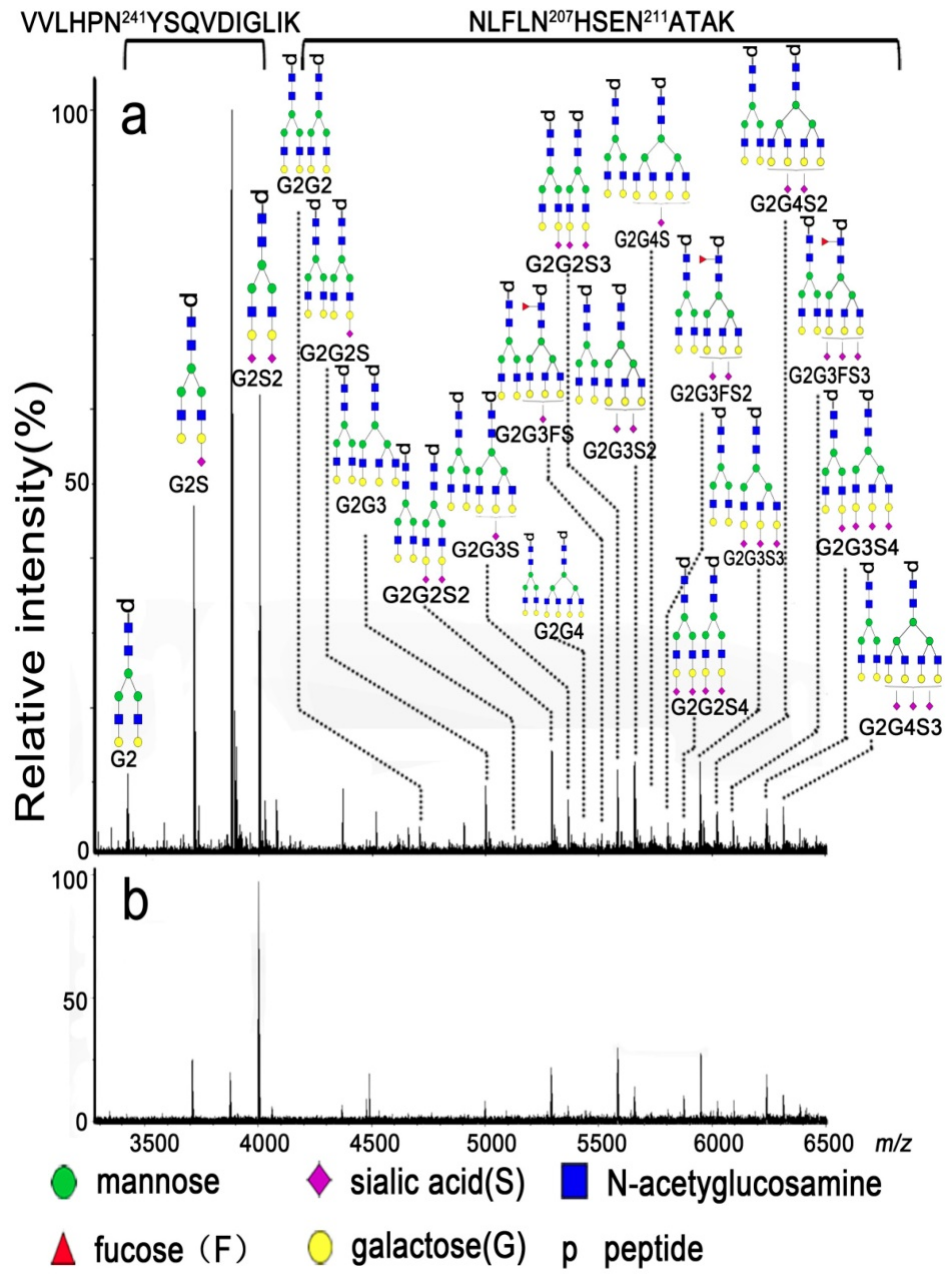
Representative mass spectra of the glycopeptides from tryptic digests of DSHp-β from one NSCLC patient (a) and one BLDs patient (b).

All mass spectra were acquired using 9.4 T Apex-ultra™ hybrid Qh-FTICR MS (Bruker Daltonics, Billerica, MA, USA) equipped with a 200 Hz, 355 nm Nd:YAG laser. The mixture of eight peptides (bombesin at *m/z* 1619.8223, ACTH_006 at *m/z* 1936.8551, ACTH_clip_18-39 at *m/z* 2465.1983, somatostatin_28 at *m/z* 3147.4710, ky_37 at *m/z* 3901.8705, dy_40 at *m/z* 4328.1557, gp_52 at *m/z* 5206.5147, ADRM at *m/z* 5969.9330, and sl_61 at *m/z* 6814.5702 in positive ion mode ) was used to calibrate the instrument over the *m/z* range of 1500-7000 in positive ion mode at the resolution of 490,000 at *m/z* 400. The dried glycopeptides were redissolved in 2 μL of ultrapure water, and 0.35 μL of the solution was spotted onto a MTP AnchorChip™ plate. After being dried at room temperature, the sample spot was overlaid by 0.35 μL of DHB solution (20 mg/mL,50% ACN (v/v) and 0.1% TFA (v/v)) and then dried again at room temperature. Mass spectra were recorded by accumulating scans at 50 laser shots per scan until the absolute intensity of the base peak reached to 1 × 10^8^. The representative mass spectra are shown in **Figure 2**.

### Data analysis

The monoisotopic mass of the detected glycopeptides from tryptic digests of DSHp-β with the signal to noise threshold of > 3.0 was extracted and transferred to Microsoft Excel. In order to reduce the effect of the amount variation of DSHp-β among patients, the relative value of each detected glycopeptide, such as G2, G2S, and G2G3S, was normalized to the total absolute intensities of all detected glycopeptides in the mass spectrum of each patient and the ratios of glycopeptides were calculated based on their corresponding relative values. Statistically different analysis between DSHp-β N-glycosylation and types of BLDs, cancer staging or age was performed by Kruskal-Wallis test. Mann-Whitney U test was used to find the correlation between N-glycopeptides (or N-glycopeptide ratios and N-glycosylation features) and the patterns, sex, or disease states. False discovery rate (FDR) controlling procedures for multi stage analysis (Benjamini-Hochberg procedure) were used to obtain the adjusted *p* value (*p*_adj_) 25 and a *p*_adj_ value of < 0.05 was considered to be statistically significant. Receiver operating characteristic (ROC) analysis was performed to calculate the area under curve (AUC), sensitivity, and specificity. The sensitivity and specificity of the biggest Youden's index was adopted 26. Correlation between the glycopeptides of DSHp-β was performed by Spearman correlation analysis and their heatmaps were constructed. Statistical analyses were carried out using SPSS software (version 16.0, SPSS, Chicago, Illinois, USA). In addition, glycosylation features were calculated as following formations: S1=G2S + G2G2S + G2G3S + G2G3FS + G2G4S, S2=G2S2 + G2G2S2 + G2G3S2 + G2G3FS2 + G2G4S2, S3=G2G2S3 + G2G3S3 + G2G3FS3 + G2G4S3, S4=G2G2S4 + G2G3S4, S=S1 + S2 + S3 + S4, and F=G2G3FS + G2G3FS2 + G2G3FS3. These symbols corresponding to their glycopeptide structures are shown in **Figure 2**.

## Results

### Patterns of serum IIRPCs of all participants

In this study, 600 of serum samples including 300 BLDs patients and 300 NSCLC patients were conducted. Serum IIRPCs, which are found to be positively correlated with pathophysiological states, were isolated using the native-PAGE (**Figure S1B**). And patterns a, b, c, d, e, f, and g of serum IIRPCs were observed in 600 serum samples, which account for 44.8%, 33.2%, 15.3%, 1.2%, 1.2%, 2.7%, and 1.7%, respectively (**Table S1** and **Figure S1A**). It should be noted that 28 of NSCLC patients and 64 of BLDs patients with the pattern c were observed. The matching factors such as age and sex between BLDs and NSCLC were considered in the following analyses, and finally the sex-and age-matched participants including 269 of NSCLC patients and 220 of BLDs patients were randomly divided into the training set including 56 of NSCLC and 56 of BLDs patients and the validation set including 213 of NSCLC and 164 of BLDs patients for DSHp-β N-glycosylation biomarker discovery. The detailed workflow chart is shown in **Figure 1**.

### Association of DSHp-β N-glycosylation with the patterns of serum IIRPCs

DSHp-β was separated from the gel bands of different types of serum IIRPCs such as patterns a, b, d, e, f, and g (**Figure S1A**). To examine the correlation of the patterns with DSHp-β N-glycosylation, we have compared the N-glycosylation between patterns a and b, and the statistical analysis for patterns d, e, f or g was not performed because of their small sample size (**Table S1**). The correlation analysis of N-glycopeptides, N-glycopeptide ratios, or N-glycosylation features of DSHp-β between patterns a and b for the age- and sex-matched BLDs or NSCLC patients was performed using Mann-Whitney U test (**Table S2**). For BLDs, different pathological state-matched data were also considered, and then FDR controlling procedures were used to obtain the adjusted *p*_adj_ value. Finally, no correlation of the patterns with DSHp-β N-glycopeptides was observed among BLDs patients, while for NSCLC patients, N-glycopeptide (G2G3FS2) was correlated with patterns (p=0.0345) (**Table S2**).

### N-glycopeptide profiling of DSHp-β

Representative mass spectra of the enriched glycopeptides of DSHp-β are shown in **Figure 2**, and the experimental *m/z* values, theoretical *m/z* values, potential glycan structure, and their corresponding peptide sequences of the detected glycopeptides of DSHp-β are listed in **Table S3**. The potential glycan structures were assigned based on their experimental masses and the GlycoMod Tool (https://web.expasy.org/glycomod/). Some of the glycan structures were confirmed using tandem mass spectra (**Figure S3**). Finally, 20 of N-glycopeptides attached to the sites of Asn241 and Asn207/211 of DSHp-β were extracted from each mass spectrum of 508 serum samples. During the entire experiment, the QC sample was analyzed once every nine test serum samples as external reference. Total 55 of mass spectra of the QC sample were obtained and the relative standard deviations (RSDs) of the glycopeptides distributed over the *m/z* range of 1500-7000 (*i.e.*, G2S2 at *m/z* 3999.8862, G2G2S at *m/z* 4995.8742, G2G2S2 at *m/z* 5287.2131, G2G2S3 at *m/z* 5578.4733, G2G3S at *m/z* 5361.2831, G2G3S2 at *m/z* 5652.6081, G2G3S3 at *m/z* 5943.8685, G2G4S2 at *m/z* 6017.8359, and G2G4S3 at *m/z* 6310.4691) from DSHp-β were calculated based on their relative values to evaluate the experimental reproducibility during the whole experiment. It is found that the RSDs of 9 of the above-mentioned representative N-glycopeptides from the QC sample were less than 20%, which is acceptable for complex biological sample analysis (**Table S4**).

### Association of DSHp-β N-glycosylation with pathological states of BLDs

In this study, we have recruited three different pathological states of BLDs including pulmonary sarcoidosis, interstitial lung disease, and pneumonia. Expression levels of N-glycopeptides, N-glycopeptide ratios, or N-glycosylation features of DSHp-β of BLDs patients with the above-mentioned three pathological states and with patterns a, b, d, e, f, and g were analyzed using Kruskal-Wallis test and significant difference among three different pathological states was not observed (*p*>0.05, **Table S5**).Therefore, all BLDs patients with three different pathological states could be put together to perform the following statistical analysis.

### Association of DSHp-β N-glycosylation with cancer staging of NSCLC patients

In this study, 48 of NSCLC patients could provide detailed information on cancer staging including stage I (6 patients), stage III (12 patients), and stage IV (30 patients). The correlation of N-glycopeptides, N-glycopeptide ratios, or N-glycosylation features of DSHp-β with cancer staging of NSCLC patients with patterns a, b, d, e, f, and g was performed using Kruskal-Wallis test, and no difference in the expression levels of DSHp-β N-glycosylation of NSCLC patients with different cancer stages was observed (*p*>0.05, **Table S6**).

### Association of DSHp-β N-glycosylation with sex

For the age-matched BLDs patients or the age-matched NSCLC patients, statistical analysis of N-glycopeptides, N-glycopeptide ratios, or N-glycosylation features of DSHp-β was performed using Mann-Whitney U test followed by FDR controlling produces. It is found that, for BLDs patients, G2G3FS3, G2G3FS/G2G3FS3, G2G3FS2/G2G3S2, and G2G3FS3/G2G3S3 are correlated with sex (*p*<0.05) and for NSCLC patients, G2G3FS3, G2G2/G2G2S3, G2G3/G2G3S4, G2G2S/G2G2S2, G2G2S/G2G2S3, and G2G3FS/G2G3S are also associated with sex. However, after FDR controlling procedures were used to obtain the adjusted *p_ad_*_j_ values, it is found that, for BLDs patients, only G2G3FS3/G2G3S3 is correlated with sex (*p*<0.05) (**Table S7**).

### Association of DSHp-β N-glycosylation with age

For the sex-matched BLDs or the sex-matched NSCLC patients, statistical analysis of N-glycopeptides, N-glycopeptide ratios, or N-glycosylation features of DSHp-β was performed using Kruskal-Wallis test (α=0.05). The correlation of age with N-glycopeptides, N-glycopeptide ratios, or N-glycosylation features of DSHp-β for NSCLC or BLDs patients was not observed (*p*_adj_>0.05, **Table S8**).

### Correlation among glycopeptides of DSHp-β

**Figure 3:**
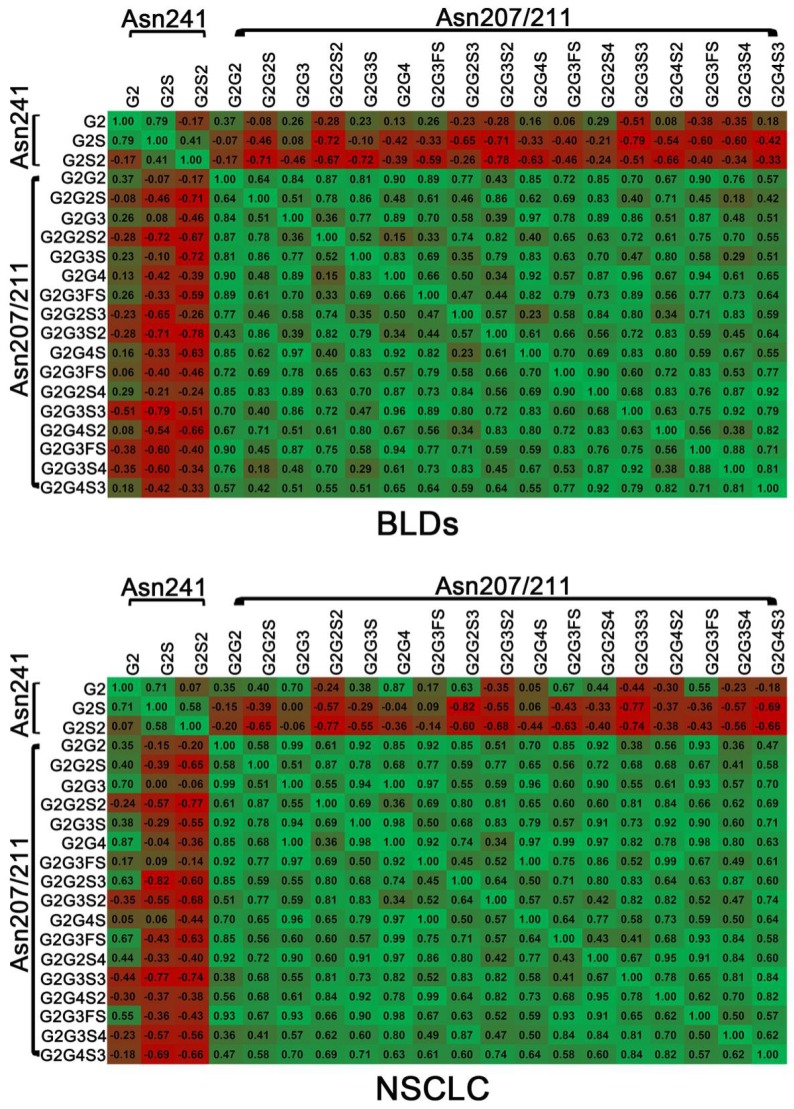
Correlation analysis between DSHp-β glycopeptides in BLDs or NSCLC patients. Green, positive correlation; red, negative correlation. The number in grid indicates the Spearman correlation coefficient.

To evaluate the correlations between different glycopeptides of DSHp-β, the correlation analysis was also performed. Positive correlations between different glycopeptides from the same site and negative correlations between different glycopeptides from different sites are observed in NSCLC patients or BLDs patients. It should be noted that positive correlations between G2 and G2S2, G2G2S, G2G2S3, or G2G3FS3 and between G2S and G2G3FS or G2G4S in NSCLC patients were detected, while inverse correlations were observed in BLDs patients and that negative correlation between G2 and G2G4S2 in NSCLC patients was detected, while inverse correlation was observed in BLDs patients (**Figure 3**).

### Change trends in DSHp-β N-glycosylation between NSCLC and BLDs patients

**Figure 4:**
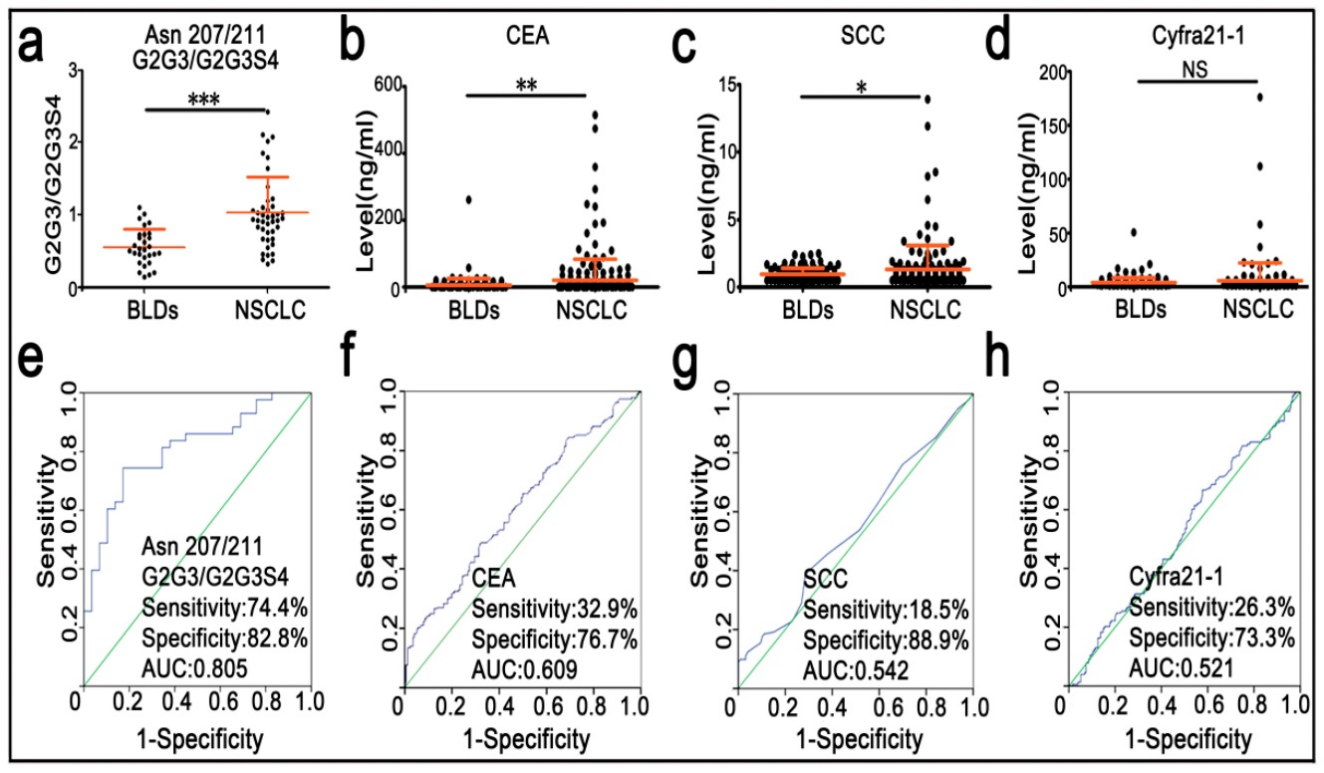
Scatter plots and diagnostic performance of serum clinical markers and G2G3/G2G3S4 at the Asn207/211 sites. *, *p* < 0.05; **, *p* < 0.01; ***, *p* < 0.001; NS, no significance.

Participants were randomly divided into the training set and the validation set. Characteristics of these participants are listed in **Table 1**. Mann-Whitney U test was used to screen differences in N-glycopeptides, N-glycopeptide ratios, or N-glycosylation features of DSHp-β between BLDs and NSCLC patients, followed by FDR controlling procedures to obtain their adjusted *p*_adj_ values. As shown in **Table 2**, the levels of G2 and G2S at the Asn241 site, G2G3S at the Asn207/211 sites, and S1 in NSCLC patients were significantly increased, while S and S2 in NSCLC patients were significantly decreased. In addition, increased monosialylation, decreased disialylation and decreased total sialylationin NSCLC were detected compared with BLDs. The ratio values of G2/G2S, G2/G2S2, and G2S/G2S2 at the Asn241 site as well as the ratio values of G2G2S2/G2G2S3, G2G3/G2G3S4, G2G3S/G2G3S2, G2G3S/G2G3S3, G2G3S/G2G3S4, G2G3S2/G2G3S3, G2G3S2/G2G3S4, G2G2S/G2G2S2, G2G2S/G2G2S3, G2G3FS/G2G3FS3, G2G4S/G2G4S3, and G2G4S2/G2G4S3 at the Asn207/211 sites in NSCLC patients were significantly increased. For the training set, ROC analysis indicates that the level ratio of G2G3/G2G3S4 at the Asn207/211 sites has a good potential to differentiate NSCLC patients from BLDs patients, with the sensitivity of 80.0%, the specificity of 63.6%, and the AUC value of 0.764. For the validation set, the level ratio of G2G3/G2G3S4 at the Asn207/211 sites is listed in **Figure 4a**. ROC analysis indicates that the ratio of G2G3/G2G3S4 at the Asn207/211 sites has a good potential to differentiate NSCLC patients from BLDs patients, with the sensitivity of 74.4%, the specificity of 82.8%, and the AUC value of 0.805 (**Figure 4e**).

### Sensitivity and specificity of serum tumor markers for differentiating BLDs from NSCLC patients

In this study, the values of serum tumor markers (CEA, SCC, and Cyfra 21-1) of 600 participants were also detected. As shown in **Figure 4b-d**, the values of CEA and SCC are significantly increased in NSCLC patients relative to BLDs patients. However, clinical significance of serum Cyfra 21-1 is not observed between BLDs and NSCLC. Based on the cut-off values of serum CEA (5.0 ng/mL), Cyfra 21-1 (3.5 ng/mL), and SCC (1.5 ng/mL), the AUC values were calculated to differentiate NSCLC from BLDs. As shown in **Figure 4f-h**, the AUC values of CEA, SCC, and Cyfra 21-1 are all lower than 0.61, with the sensitivity of <33% and the specificity of <89%.

## Discussion

Previous study found that the levels of total fucosylated di-, tri- andtetra-branched glycans of Hp-β increased in sera of patients with pancreatic cancer using a strategy of SDS-PAGE separation with mass spectrometry detection 12. Different lectin blot arrays indicated that serum Hp-β of patients with colon cancer significantly increased compared with that of healthy subjects 27. And change in glycosylation of serum Hp-β was closely associated with chronic hepatitis C, hepatitis C, induced liver cirrhosis, and hepatocellular carcinoma 28.

Lectin array also revealed that hypersialylated fucosylated and hyposialylated fucosylated species of Hp were associated with patients with hepatocellular carcinoma 29. Liquid chromatography-mass spectrometry-based method demonstrated that significantly increased sialylation at Asn207 and Asn211 sites of serum Hp-β and the fucosylated glycoforms in patients with liver cirrhosis and hepatocellular carcinoma were observed compared with those in hepatitis B virus patients and healthy subjects 16. Glycomics-based approach found that serum fucosylated Hp of lung cancer significantly increased compared to healthy controls 15. In our study, comparison of N-glycosylation of DSHp-β, one of the major components of serum IIRPCs that were closely associated with the disease states 22, between NSCLC patients and BLDs patients was only performed because of the lack of serum IIRPCs for healthy controls. So, our study is the first time to perform the comparison of DSHp-β glycosylation between BLDs and NSCLC. In addition, in our study, the intact glycopeptides of DSHp-β with site information have been analyzed to obtain the microheterogeneity of DSHp-β glycosylation.

Based on the levels of serum IIRPCs, we could distinguish individuals with pathophysiological states from healthy states 22, and in term of the N-glycosylation features of DSHp-β, malignant diseases and benign diseases could be diagnosed with high accuracy. In our study, we found that the total sialylation level of DSHp-β in BLDs was significantly increased compared with NSCLC, while the monosialylation of DSHp-β in NSCLC patients were significantly increased relative to BLDs patients.

These findings indicate that different sialylation levels of the DSHp-β between NSCLC and BLDs patients might be associated with their individual pathological states and that different mechanisms of the inflammatory response between NSCLC and BLDs patients might occur. In addition, as shown in **Table 2**, the ratio of G2G3/G2G3S4 at the sites of Asn207/211 of DSHp-β in BLDs (0.537) was much lower than that in NSCLC (1.0273), suggesting that fully sialylated glycan at the Asn207/211 in NSCLC decreased relative to BLDs. Previous studies indicated that the increased level of sialylation at the sites of Asn207/211 of total serum Hp in patients with liver cirrhosis and hepatocellular carcinoma was detected compared with those in patients with hepatitis B virus and healthy individuals 16 and that the increased level of sialylation of total serum Hp in hepatocellular carcinoma patient was observed relative to patients with hepatitis C viral infection and chronic hepatitis C 28. In addition, the glycopeptides of serum total Hp indicated that the site-specific N-glycopeptides at the sites of Asn184 and Asn211 in early hepatocellular carcinoma significantly increased compared to cirrhosis (p < 0.05) and normal controls (p ≤ 0.001)^17^. It should be noted that the difference between our study and previous studies may be due to the difference between the ratios of glycopeptides at the sites of Asn207/211 of DSHp-β and the values of glycopeptides at the sites of Asn207/211 of serum total Hp-β. Our findings may provide new insight to understand the correlation of inflammation with change in sialylation of DSHp-β. In addition, the correlation analysis has shown no correlation between the changes in DSHp-β glycopeptides and different staging of NSCLC (**Table S6**), suggesting that DSHp-β glycosylation was associated with pathophysiological states and no correlation between different cancer staging of NSCLC. Our results are similar to the previous findings 30.

ROC analysis indicated that the level ratio of G2G3/G2G3S4 at the Asn207 and Asn211 sites of DSHp-β has a better capability to differentiate NSCLC patients from BLDs patients, with the sensitivity of 74.4%, the specificity of 82.8% and the AUC value of 0.805 compared with serum tumor markers (such as CEA, SCC, and Cyfra 21-1), these data again imply that the sialylation level of DSHp-β could reflect differences in pathological states between NSCLC and BLDs patients.

## Conclusions

In this study, we applied a strategy developed by our laboratory to obtain DSHp-β from complex biological serum samples. This is the first time to study change in the sialylation of DSHp-β between NSCLC and BLDs. Changes in the sialylation levels of DSHp-β between NSCLC and BLDs can reflect difference in the pathophysiological states and in the mechanisms of inflammatory response. The glycosylation of DSHp-β can also distinguish NSCLC from BLDs patients with high diagnostic accuracy compared with current clinical available serum markers.

## Supplementary Material

Supplementary figures and tables.

## Figures and Tables

**Table 1:** Clinical characteristics of the 508 patients with BLDs or NSCLC

	Pathological states	Sex (M/F)	Age (mean ± SD, range)	BLDs				NSCLC		
				Pulmonary sarcoidosis	Pneumonia	Interstitial lung disease		Adenocarcinoma	Squamous cell carcinoma	Unknown subtypes of NSCLC
**Participants**	**BLDs**	113/123	56.81 ± 9.57, 30-80	161	35	40		/	/	/
	**NSCLC**	136/136	57.6 4± 8.88, 33-85	/	/	/		51	20	201
										
**Training set**	**BLDs**	28/28	57.16 ± 9.28, 33-77	38	6	12		/	/	/
	**NSCLC**	28/28	57.78 ± 10.47, 33-80	/	/	/		8	5	43
										
**Validation set**	**BLDs**	76/88	56.9 2 ± 9.38, 30-74	109	27	28		/	/	/
	**NSCLC**	108/105	57.58 ± 8.42, 35-74	/	/	/		40	15	158

BLDs, benign lung diseases; NSCLC, non-small cell lung cancer; M, male; F, female.

**Table 2:** Comparison of N-glycopeptides, N-glycopeptide ratios, and N-glycosylation features of DSHp-β between BLDs and NSCLC patients.

Glycopeptides, glycopeptide ratios, or glycosylation features	Training set					Validation set			
	BLDs	NSCLC	*p* value	*p_adj_*value		BLDs	NSCLC	*p* value	*p_adj_*value
**VVLHPN_241_YSQVDIGLIK**									
**G2**	0.037±0.031	0.072±0.047	<0.001	<0.001		0.046±0.045	0.061±0.040	<0.001	<0.001
**G2S**	0.170±0.067	0.224±0.116	0.015	0.033		0.188±0.095	0.234±0.112	<0.001	<0.001
**G2/G2S**	0.195±0.146	0.271±0.161	<0.001	0.002		0.206±0.128	0.239±0.119	<0.001	<0.001
**G2/G2S2**	0.105±0.148	0.423±0.791	<0.001	<0.001		0.120±0.088	0.231±0.257	<0.001	<0.001
**G2S/G2S2**	0.433±0.145	0.985±0.930	<0.001	<0.001		0.559±0.219	0.818±0.437	<0.001	<0.001
**NLFLN_207_HSEN_211_ATAK**									
**G2G3S**	0.028±0.037	0.059±0.046	<0.001	<0.001		0.038±0.042	0.047±0.050	0.006	0.011
**G2G2S2/G2G2S3**	0.863±0.218	1.222±0.524	0.001	0.003		1.126±0.385	1.400±0.572	<0.001	<0.001
**G2G3/G2G3S4**	0.361±0.198	0.953±0.484	0.021	0.043		0.537±0.260	1.027±0.501	<0.001	<0.001
**G2G3S/G2G3S2**	0.584±0.242	0.884±0.574	0.004	0.011		0.550±0.167	0.703±0.295	<0.001	<0.001
**G2G3S/G2G3S3**	0.388±0.180	0.764±0.364	<0.001	<0.001		0.492±0.234	0.867±0.509	<0.001	<0.001
**G2G3S/G2G3S4**	0.536±0.509	0.953±0.495	<0.001	0.001		0.767±0.421	1.431±1.253	0.001	0.002
**G2G3S2/G2G3S3**	0.637±0.139	1.046±0.398	<0.001	<0.001		0.844±0.288	1.145±0.424	<0.001	<0.001
**G2G3S2/G2G3S4**	0.913±0.594	1.375±0.843	0.003	0.011		1.334±0.652	1.908±1.244	0.001	0.002
**G2G2S/G2G2S2**	0.443±0.124	0.816±0.424	<0.001	<0.001		0.534±0.151	0.750±0.294	<0.001	<0.001
**G2G2S/G2G2S3**	0.391±0.150	1.024±0.617	<0.001	<0.001		0.645±0.329	1.115±0.703	<0.001	<0.001
**G2G3FS/G2G3FS3**	0.564±0.187	0.952±0.174	0.004	0.011		0.753±0.244	0.936±0.309	0.003	0.006
**G2G4S/G2G4S3**	0.607±0.202	0.910±0.293	0.009	0.020		0.671±0.196	1.065±0.390	<0.001	<0.001
**G2G4S2/G2G4S3**	0.863±0.168	1.142±0.328	0.006	0.015		0.970±0.260	1.239±0.451	<0.001	<0.001
**S1**	0.216±0.062	0.319±0.139	<0.001	<0.001		0.255±0.103	0.325±0.121	<0.001	<0.001
**S2**	0.500±0.099	0.422±0.125	<0.001	<0.001		0.465±0.090	0.448±0.097	0.025	0.043
**S**	0.954±0.044	0.895±0.105	<0.001	<0.001		0.951±0.069	0.919±0.078	<0.001	<0.001

## References

[1] Torre LA, Siegel RL, Jemal A (2016). Lung Cancer Statistics. Adv Exp Med Biol.

[2] Chen W, Zheng R, Baade PD, Zhang S, Zeng H, Bray F, Jemal A, Yu XQ, He J (2016). Cancer statistics in China, 2015. CA Cancer J Clin.

[3] Herbst RS, Heymach JV, Lippman SM (2008). Lung cancer. N Engl J Med.

[4] Iachina M, Jakobsen E, Fallesen AK, Green A (2017). Transfer between hospitals as a predictor of delay in diagnosis and treatment of patients with Non-Small Cell Lung Cancer - a register based cohort-study. BMC Health Serv Res.

[5] Rolston KV, Rodriguez S, Dholakia N, Whimbey E, Raad I (1997). Pulmonary infections mimicking cancer: a retrospective, three-year review. Support Care Cancer.

[6] Ost D, Fein AM, Feinsilver SH (2003). Clinical practice. The solitary pulmonary nodule. N Engl J Med.

[7] Pak K, Park S, Cheon GJ, Kang KW, Kim IJ, Lee DS, Kim EE, Chung JK (2015). Update on nodal staging in non-small cell lung cancer with integrated positron emission tomography/computed tomography: a meta-analysis. Ann Nucl Med.

[8] Dincer HE (2013). Linear EBUS in staging non-small cell lung cancer and diagnosing benign diseases. J Bronchology Interv Pulmonol.

[9] Lowe JB (2003). Glycan-dependent leukocyte adhesion and recruitment in inflammation. Curr Opin Cell Biol.

[10] Pearce OM, Laubli H (2016). Sialic acids in cancer biology and immunity. Glycobiology.

[11] Quaye IK (2008). Haptoglobin, inflammation and disease. Trans R Soc Trop Med Hyg.

[12] Nakano M, Nakagawa T, Ito T, Kitada T, Hijioka T, Kasahara A, Tajiri M, Wada Y, Taniguchi N, Miyoshi E (2008). Site-specific analysis of N-glycans on haptoglobin in sera of patients with pancreatic cancer: a novel approach for the development of tumor markers. Int J Cancer.

[13] Zhang S, Shang S, Li W, Qin X, Liu Y (2016). Insights on N-glycosylation of human haptoglobin and its association with cancers. Glycobiology.

[14] Varadi C, Mittermayr S, Szekrenyes A, Kadas J, Takacs L, Kurucz I, Guttman A (2013). Analysis of haptoglobin N-glycome alterations in inflammatory and malignant lung diseases by capillary electrophoresis. Electrophoresis.

[15] Tsai HY, Boonyapranai K, Sriyam S, Yu CJ, Wu SW, Khoo KH, Phutrakul S, Chen ST (2011). Glycoproteomics analysis to identify a glycoform on haptoglobin associated with lung cancer. Proteomics.

[16] Zhang S, Jiang K, Sun C, Lu H, Liu Y (2013). Quantitative analysis of site-specific N-glycans on sera haptoglobin beta chain in liver diseases. Acta Biochim Biophys Sin (Shanghai).

[17] Zhu J, Chen Z, Zhang J, An M, Wu J, Yu Q, Skilton S, Bern M, Sen K, Li L, Lubman D (2019). Differential Quantitative Determination of Site-Specific Intact N-Glycopeptides in Serum Haptoglobin between Hepatocellular Carcinoma and Cirrhosis Using LC-EThcD-MS/MS. J Proteome Res.

[18] Lee J, Hua S, Lee S, Oh M, Yun J, Kim J, Kim J, Kim J, An H (2018). Designation of fingerprint glycopeptides for targeted glycoproteomic analysis of serum haptoglobin: insights into gastric cancer biomarker discovery. Anal Bioanal Chem.

[19] Wang D, Hincapie M, Rejtar T, Karger BL (2011). Ultrasensitive characterization of site-specific glycosylation of affinity-purified haptoglobin from lung cancer patient plasma using 10 mum i.d. porous layer open tubular liquid chromatography-linear ion trap collision-induced dissociation/electron transfer dissociation mass spectrometry. Anal Chem.

[20] Song G, Liu Y, Wang Y, Ren G, Guo S, Ren J, Zhang L, Li Z (2015). Personalized biomarkers to monitor disease progression in advanced non-small-cell lung cancer patients treated with icotinib. Clin Chim Acta.

[21] Liu Y, Zhang D, Cheng Y, Li Z (2015). Elevated serum immunoinflammation-related protein complexes are associated with psychosis. Psychiatry Res.

[22] Wang Y, Song G, Wang Y, Qiu L, Qin X, Liu H, Li F, Wang X, Li F, Guo S, Zhang Y, Li Z (2014). Elevated serum levels of circulating immunoinflammation-related protein complexes are associated with cancer. J Proteome Res.

[23] Ferens-Sieczkowska M, Kratz EM, Kossowska B, Passowicz-Muszynska E, Jankowska R (2013). Comparison of haptoglobin and alpha(1)-acid glycoprotein glycosylation in the sera of small cell and non-small cell lung cancer patients. Postepy Hig Med Dosw (Online).

[24] Zhang M, Liu Y, Zhang D, Chen T, Li Z (2017). Facile and Selective Enrichment of Intact Sialoglycopeptides Using Graphitic Carbon Nitride. Anal Chem.

[25] Benjamini Y, Hochberg Y (1995). Controlling the False Discovery Rate: A Practical and Powerful Approach to Multiple Testing. J Royal Statistical Soc. Series B (Methodological).

[26] Hilden J, Glasziou P (1996). Regret graphs, diagnostic uncertainty and Youden's Index. Statistics in medicine.

[27] Park SY, Yoon SJ, Jeong YT, Kim JM, Kim JY, Bernert B, Ullman T, Itzkowitz SH, Kim JH, Hakomori SI (2010). N-glycosylation status of beta-haptoglobin in sera of patients with colon cancer, chronic inflammatory diseases and normal subjects. Int J Cancer.

[28] Mondal G, Saroha A, Bose PP, Chatterjee BP (2016). Altered glycosylation, expression of serum haptoglobin and alpha-1-antitrypsin in chronic hepatitis C, hepatitis C induced liver cirrhosis and hepatocellular carcinoma patients. Glycoconj J.

[29] Ang IL, Poon TC, Lai PB, Chan AT, Ngai SM, Hui AY, Johnson PJ, Sung JJ (2006). Study of serum haptoglobin and its glycoforms in the diagnosis of hepatocellular carcinoma: a glycoproteomic approach. J Proteome Res.

[30] Hoagland LFt, Campa MJ, Gottlin EB, Herndon JE 2nd, Patz EF Jr (2007). Haptoglobin and posttranslational glycan-modified derivatives as serum biomarkers for the diagnosis of nonsmall cell lung cancer. Cancer.

