# Supplementary material for: Disease-specific haptoglobin-β chain N-glycosylation as biomarker to differentiate non-small cell lung cancer from benign lung diseases
Source: J Cancer. 2019 Sep 7;10(23):5628–37. doi: 10.7150/jca.32690 (PMC6843889; doi:10.7150/jca.32690)
Supplement: Supplementary file 1 — Supplementary figures and tables. [file jcav10p5628s1.pdf]

Supporting Information

Disease-specific haptoglobin-β chain N-glycosylation as biomarker to differentiate non-small cell lung cancer from benign lung diseases

Tianjing Chen[1], Chengyan He[2], Mo Zhang[1], Xiaoou Li[3], Xiaofeng Liu[3], Yujie Liu[1], Dan Zhang[1], and Zhili Li[1,*]

1. Department of Biophysics and Structural Biology, Institute of Basic Medical Sciences, Chinese Academy of Medical Sciences & School of Basic Medicine, Peking Union Medical College, Beijing, PR China

2. Clinical Lab Diagnosis, China-Japan Union Hospital, Jilin University, Changchun, PR China

3. Department of Laboratory, Tumor Hospital of Jilin Province, Changchun, PR China

***Corresponding author**: Zhili Li, Department of Biophysics and Structural Biology, Institute of Basic Medical Sciences, Chinese Academy of Medical Sciences & School of Basic Medicine, Peking Union Medical College, 5 Dongdan San Tiao, Beijing 100005, PR China

Tel/Fax: +86-10-69156479

E-mail: lizhili@ibms.pumc.edu.cn

Table S1. Patterns of serum IIRPCs of patients with NSCLC and BLDs

	a	b	c	d	e	f	g
BLDs	122	88	64	3	4	11	8
NSCLC	147	111	28	4	3	5	2
Total	269	199	92	7	7	16	10
Percentage	44.8%	33.2%	15.3%	1.2%	1.2%	2.7%	1.7%

Percentage of cases (x)=number(x)/number(a + b + c + d + e + f + g); BLDs, benign lung diseases; NSCLC, non-small cell lung cancer.

Table S2. Comparison of the N-glycopeptides, N-glycopeptide ratios, and N-glycosylation features of DSHp-β between patterns a and b in BLDs or NSCLC patients.

**N-glycopeptides, N-glycopeptide ratios and N-glycosylation features**	**BLDs**		**NSCLC**	
	***p* value**	**p_{adj} value**	***p* value**	**p_{adj} value**
**VVLHPN$_{241}$YSQVDIGLIK**				
G2	***0.019***	0.147	0.789	0.891
G2S	0.965	1.015	0.278	0.679
G2S2	***0.028***	0.188	0.305	0.715
G2/G2S	***0.004***	0.113	0.153	0.585
G2/G2S2	***0.005***	0.096	0.575	0.856
G2S/G2S2	***0.049***	0.211	0.850	0.894
**NLFLN$_{207}$HSEN$_{211}$ATAK**				
G2G2	0.349	0.626	0.093	0.434
G2G2S	0.455	0.711	0.210	0.581
G2G3	0.764	0.863	***0.019***	0.288
G2G2S2	0.228	0.479	0.593	0.861
G2G3S	0.133	0.368	0.209	0.608
G2G4	1.000	1.017	***0.042***	0.423
G2G3FS	0.412	0.680	0.112	0.456
G2G2S3	0.531	0.705	0.568	0.888
G2G3S2	0.089	0.300	0.328	0.689
G2G4S	0.414	0.664	0.188	0.572
G2G3FS2	0.092	0.295	***0.001***	***0.035***
G2G2S4	0.486	0.722	0.391	0.746
G2G3S3	0.179	0.455	0.867	0.881
G2G4S2	0.958	1.025	0.370	0.728
G2G3FS3	0.872	0.967	0.176	0.564
G2G3S4	0.610	0.715	0.811	0.900
G2G4S3	***0.048***	0.224	0.064	0.392
G2G2/G2G2S2	***0.009***	0.107	0.820	0.893
G2G2S2/G2G2S3	0.050	0.204	0.484	0.819
G2G2/G2G2S	***0.036***	0.200	0.613	0.814
G2G2/G2G2S3	0.206	0.465	0.707	0.845
G2G2/G2G2S4	0.309	0.589	0.659	0.856
G2G3/G2G3S	0.599	0.730	0.820	0.878
G2G3/G2G3S2	***0.037***	0.189	0.367	0.746
G2G3/G2G3S3	0.515	0.698	0.780	0.898
G2G3/G2G3S4	0.554	0.704	***0.042***	0.363
G2G3S/G2G3S2	0.498	0.723	0.855	0.884
G2G3S/G2G3S3	0.285	0.580	0.166	0.597

G2G3S/G2G3S4	0.130	0.378	0.233	0.618
G2G3S2/G2G3S3	0.189	0.443	0.448	0.805
G2G3S2/G2G3S4	0.183	0.448	0.700	0.853
G2G3S3/G2G3S4	0.168	0.445	0.400	0.739
G2G2S/G2G2S2	0.966	0.999	0.171	0.579
G2G2S/G2G2S3	0.208	0.453	0.272	0.692
G2G2S/G2G2S4	0.109	0.333	0.713	0.837
G2G2S2/G2G2S4	0.003	0.186	0.603	0.836
G2G2S3/G2G2S4	0.053	0.204	0.096	0.417
G2G3FS/G2G3FS2	***0.009***	0.081	0.941	0.941
G2G3FS/G2G3FS3	0.079	0.284	0.601	0.853
G2G3FS2/G2G3FS3	0.499	0.708	0.456	0.795
G2G4/G2G4S	0.564	0.702	0.016	0.320
G2G4/G2G4S2	1.000	1.000	0.044	0.297
G2G4/G2G4S3	0.380	0.662	0.025	0.307
G2G4S/G2G4S2	0.759	0.874	0.487	0.803
G2G4S/G2G4S3	0.553	0.717	0.305	0.689
G2G4S2/G2G4S3	0.505	0.700	0.611	0.829
G2G3FS/G2G3S	***0.009***	0.089	0.571	0.871
G2G3FS2/G2G3S2	0.408	0.691	0.067	0.374
G2G3FS3/G2G3S3	0.299	0.588	***0.015***	0.471
S1	0.602	0.720	0.666	0.846
S2	***0.009***	0.131	0.314	0.685
S3	0.457	0.696	0.526	0.844
S4	0.909	0.990	0.697	0.868
S	***0.034***	0.210	0.088	0.448
F	0.346	0.639	***0.043***	0.324

A p value of <0.05 was considered to be statistically significant.

BLDs patients:

pattern a: sex(M/F), 24/24; age(mean, range), 55.0,30-70 (pulmonary sarcoidosis (n=32), pneumonia (n=8), and interstitial lung disease(n=8)).

pattern b: sex(M/F):24/25, age(mean, range):56.9,31-73 (pulmonary sarcoidosis (n=33), pneumonia(n=7), and interstitial lung disease(n=9)).

NSCLC patients:

pattern a: sex(M/F), 73/74; age(mean, range), 57.3,33-77.

Pattern b: sex(M/F), 54/56; age(mean, range), 58.1,35-74.

M: male; F: female.

Table S3. The detected N-glycopeptides of DSHp-β and their potential glycan structures

Glycopeptides	Experimental *m/z*	Theoretical m/z	Error (Da)	Accuracy (ppm)	potential glycan structure/mass	Glycan formula
**VVLHPN$_{241}$YSQVDIGLIK**						
**G2**	3417.5018	3418.6958	-1.194	-349.26	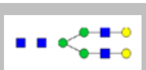 1640.5922 Da	(Hex)2 (HexNAc)2 + (Man)3(GlcNAc)2
**G2S**	3708.6629	3709.7912	-1.1283	-304.14	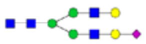 1931.6875 Da	(Hex)2 (HexNAc)2 (NeuAc)1 + (Man)3(GlcNAc)2
**G2S2**	3999.8862	4000.8866	-1.0004	-250.04	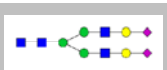 2222.7829 Da	(Hex)2 (HexNAc)2 (NeuAc)2 + (Man)3(GlcNAc)2
**NLFLN$_{207}$HSEN$_{211}$ATAK**						
**G2G2**	4704.0852	4704.7606	-0.6754	-143.56	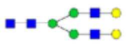 1640.5922 Da<br>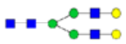 1640.5922 Da	[(Hex)2 (HexNAc)2 +(Man)3 (GlcNAc)2] + [(Hex)2 (HexNAc)2 +(Man)3 (GlcNAc)2]
**G2G2S**	4995.8742	4995.856	0.0182	3.64	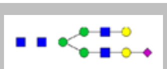 1931.6875 Da<br>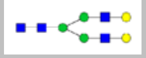 1640.5922 Da	[(Hex)2 (HexNAc)2 (NeuAc)1 +(Man)3 (GlcNAc)2] + [(Hex)2 (HexNAc)2 +(Man)3 (GlcNAc)2]
**G2G2S2**	5287.2131	5286.9514	0.2617	49.50	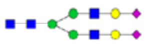 2222.7829 Da<br>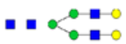 1640.5922 Da	[(Hex)2 (HexNAc)2(NeuAc)2 +(Man)3 (GlcNAc)2] + [(Hex)2 (HexNAc)2 +(Man)3 (GlcNAc)2]

**G2G2S3**	5578.4733	5578.0468	0.4265	76.46	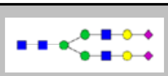 2222.7829 Da<br>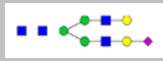 1931.6875 Da	[(Hex)2 (HexNAc)2 (NeuAc)2 +(Man)3 (GlcNAc)2] + [(Hex)2 (HexNAc)2 (NeuAc)1 +(Man)3 (GlcNAc)2]
**G2G2S4**	5869.7643	5869.1422	0.6221	106.00	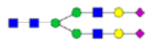 2222.7829 Da<br>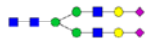 2222.7829 Da	[(Hex)2 (HexNAc)2 (NeuAc)2 +(Man)3 (GlcNAc)2] + [(Hex)2 (HexNAc)2 (NeuAc)2 +(Man)3 (GlcNAc)2]
**G2G3S**	5361.2831	5360.9882	0.2949	55.01	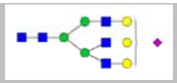 2296.8198 Da<br>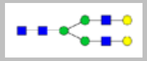 1640.5922 Da	[(Hex)3 (HexNAc)3 (NeuAc)1 +(Man)3 (GlcNAc)2] + [(Hex)2 (HexNAc)2 +(Man)3 (GlcNAc)2]
**G2G3**	5070.9386	5069.8928	1.0458	206.28	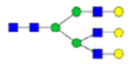 2005.7244 Da<br>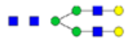 1640.5922 Da	[(Hex)3 (HexNAc)3 +(Man)3 (GlcNAc)2] + [(Hex)2 (HexNAc)2 +(Man)3 (GlcNAc)2]
**G2G3S3**	5943.8685	5943.179	0.6895	116.02	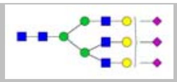 2879.0106 Da<br>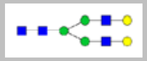 1640.5922 Da	[(Hex)3 (HexNAc)3 (NeuAc)3 +(Man)3 (GlcNAc)2] + [(Hex)2 (HexNAc)2 +(Man)3 (GlcNAc)2]
**G2G3S4**	6235.3923	6234.2744	1.1179	179.32	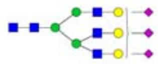 2879.0106 Da<br>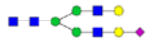 1931.6875 Da	[(Hex)3 (HexNAc)3 (NeuAc)3 +(Man)3 (GlcNAc)2] + [(Hex)2 (HexNAc)2 (NeuAc)1+(Man)3 (GlcNAc)2]
**G2G3FS**	5507.3909	5507.0461	0.3448	62.61	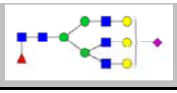 2442.8777Da	[(Hex)3 (HexNAc)3 (NeuAc)1 (Fuc)1 + (Man)3 (GlcNAc)2] + [(Hex)2 (HexNAc)2 + (Man)3(GlcNAc)2]

					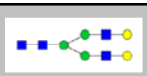 1640.5922 Da	
**G2G3FS2**	5798.4513	5798.1415	0.3098	53.43	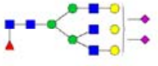 2733.9731 Da<br>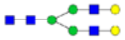 1640.5922 Da	[(Hex)3 (HexNAc)3 (NeuAc)2 (Fuc)1 + (Man)3 (GlcNAc)2] + [(Hex)2 (HexNAc)2 + (Man)3(GlcNAc)2]
**G2G3FS3**	6090.1032	6089.2369	0.8663	142.27	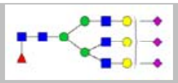 3025.0685 Da<br>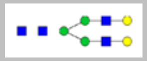 1640.5922 Da	[(Hex)3 (HexNAc)3 (NeuAc)3 (Fuc)1 + (Man)3 (GlcNAc)2] + [(Hex)2 (HexNAc)2 + (Man)3(GlcNAc)2]
**G2G4**	5435.4077	5435.025	0.3827	70.41	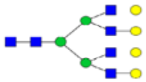 2370.8566 Da<br>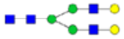 1640.5922 Da	[(Hex)4 (HexNAc)4 +(Man)3 (GlcNAc)2] + [(Hex)2 (HexNAc)2 +(Man)3 (GlcNAc)2]
**G2G4S**	5726.7593	5726.1204	0.6389	111.58	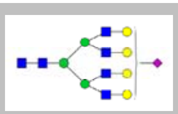 2661.9520 Da<br>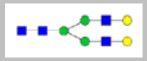 1640.5922 Da	[(Hex)4 (HexNAc)4 (NeuAc)1 +(Man)3 (GlcNAc)2] + [(Hex)2 (HexNAc)2 +(Man)3 (GlcNAc)2]
**G2G4S2**	6017.8359	6017.2158	0.6201	103.05	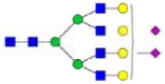 2953.0474 Da<br>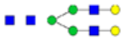 1640.5922 Da	[(Hex)4 (HexNAc)4 (NeuAc)2 +(Man)3 (GlcNAc)2] + [(Hex)2 (HexNAc)2 +(Man)3 (GlcNAc)2]

**G2G4S3**	6310.4691	6308.3112	2.1579	342.07	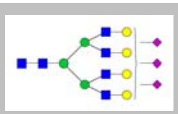<br>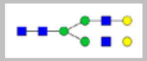	3244.1428 Da<br>1640.5922 Da	[(Hex)4 (HexNAc)4 (NeuAc)3 +(Man)3 (GlcNAc)2] + [(Hex)2 (HexNAc)2 +(Man)3 (GlcNAc)2]

Table S4. The relative standard deviations (RSDs) of 9 DSHp-β N-glycopeptides of the QC sample as external reference.

**Glycosylation site**	**Asn241**	**Asn207/211**							
**Glycopeptide**	G2S2	G2G2S	G2G2S2	G2G2S3	G2G3S	G2G3S2	G2G3S3	G2G4S2	G2G4S3
m/z	3999.8862	4995.8742	5287.2131	5578.4733	5361.2831	5652.6081	5943.8685	6017.8359	6310.4691
**RSD (%)**	19.46	19.92	15.41	18.28	19.5	16.58	16.53	19.49	19.53

Table S5. Comparison of the N-glycopeptides, N-glycopeptide ratios, and N-glycosylation features of DSHp-β between three different pathological states of BLDs patients with patterns a, b, d, e, f, and g.

N-glycopeptides, N-glycopeptide ratios and N-glycosylation features	*p* value
**VVLHPN$_{241}$YSQVDIGLIK**	
G2	0.892
G2S	0.973
G2S2	0.340
G2/G2S	0.973
G2/G2S2	0.943
G2S/G2S2	0.999
**NLFLN$_{207}$HSEN$_{211}$ATAK**	
G2G2	0.698
G2G2S	0.143
G2G3	0.710
G2G2S2	0.858
G2G3S	0.487
G2G4	0.644
G2G3FS	0.102
G2G2S3	0.871
G2G3S2	0.355
G2G4S	0.814
G2G3FS2	0.361
G2G2S4	0.859
G2G3S3	0.789
G2G4S2	0.755
G2G3FS3	0.460
G2G3S4	0.161
G2G4S3	0.239
G2G2/G2G2S2	0.313
G2G2S2/G2G2S3	0.060
G2G2/G2G2S	0.821
G2G2/G2G2S3	0.120
G2G2/G2G2S4	0.186
G2G3/G2G3S	0.248
G2G3/G2G3S2	0.802
G2G3/G2G3S3	0.928
G2G3/G2G3S4	0.540
G2G3S/G2G3S2	0.729
G2G3S/G2G3S3	0.370
G2G3S/G2G3S4	0.745
G2G3S2/G2G3S3	0.138

**N-glycopeptides, N-glycopeptide ratios and N-glycosylation features**	***p* value**
G2G3S2/G2G3S4	0.242
G2G3S3/G2G3S4	0.252
G2G2S/G2G2S2	0.285
G2G2S/G2G2S3	0.142
G2G2S/G2G2S4	0.431
G2G2S2/G2G2S4	0.458
G2G2S3/G2G2S4	0.933
G2G3FS/G2G3FS2	0.101
G2G3FS/G2G3FS3	0.206
G2G3FS2/G2G3FS3	0.981
G2G4/G2G4S	0.222
G2G4/G2G4S2	0.258
G2G4/G2G4S3	0.936
G2G4S/G2G4S2	0.980
G2G4S/G2G4S3	0.690
G2G4S2/G2G4S3	0.876
G2G3FS/G2G3S	0.959
G2G3FS2/G2G3S2	0.251
G2G3FS3/G2G3S3	0.169
S1	0.508
S2	0.719
S3	0.856
S4	0.250
S	0.671
F	0.650

Three different pathological states of BLDs:

pulmonary sarcoidosis, sex (M/F): 76/85, age (mean, range):56.4, 31-80;

Pneumonia: sex (M/F):16/19, age (mean, range):56.6, 37-77;

Interstitial lung disease: sex (M/F):21/19, age (mean, range):58.8, 30-74.

Statistical analysis was performed by Kruskal-Wallis test (α=0.05).
M: male; F: female.

Table S6. Comparison of N-glycopeptides, N-glycopeptide ratios, and N-glycosylation features of DSHp-β between different staging of NSCLC patients.

N-glycopeptides, N-glycopeptide ratios, and N-glycosylation features	*p* value	p_{adj} value
**VVLHPN$_{241}$YSQVDIGLIK**		
G2	0.910	0.994
G2S	0.942	0.992
G2S2	0.114	1.677
G2/G2S	0.533	0.767
G2/G2S2	0.421	0.856
G2S/G2S2	0.708	0.853
**NLFLN$_{207}$HSEN$_{211}$ATAK**		
G2G2	0.934	1.002
G2G2S	0.191	1.409
G2G2S2	0.294	1.444
G2G2S3	0.517	0.762
G2G2S4	0.727	0.858
G2G3	0.692	0.851
G2G3S	0.322	1.056
G2G3S2	***0.003***	0.183
G2G3S3	0.267	1.431
G2G3S4	0.435	0.754
G2G4	0.168	1.649
G2G4S	0.229	1.351
G2G4S2	0.318	1.250
G2G4S3	0.430	0.818
G2G3FS	0.646	0.847
G2G3FS2	0.443	0.688
G2G3FS3	0.322	1.186
G2G2/G2G2S	0.331	1.027
G2G2/G2G2S2	0.635	0.851
G2G2/G2G2S3	0.311	1.312
G2G2S/G2G2S2	0.657	0.843
G2G2S/G2G2S3	0.362	1.018
G2G2S/G2G2S4	0.442	0.705
G2G2S2/G2G2S3	0.302	1.368
G2G2S2/G2G2S4	0.378	0.970
G2G2S3/G2G2S4	0.091	1.796
G2G3/G2G3S	0.901	1.003
G2G3/G2G3S2	0.405	0.920
G2G3/G2G3S3	0.506	0.766
G2G3/G2G3S4	0.439	0.739
G2G3FS/G2G3FS2	0.751	0.869

**N-glycopeptides, N-glycopeptide ratios, and N-glycosylation features**	***p* value**	**p_{adj} value**
G2G3FS/G2G3FS3	0.607	0.852
G2G3FS/G2G3S	0.633	0.868
G2G3FS2/G2G3FS3	0.228	1.491
G2G3FS2/G2G3S2	0.134	1.577
G2G3FS3/G2G3S3	0.965	0.982
G2G3S/G2G3S2	0.431	0.794
G2G3S/G2G3S3	0.322	1.117
G2G3S/G2G3S4	0.351	1.035
G2G3S2/G2G3S3	0.071	2.080
G2G3S2/G2G3S4	0.184	1.547
G2G3S3/G2G3S4	0.402	0.948
G2G4/G2G4S2	0.407	0.889
G2G4/G2G4S3	0.368	0.987
G2G4S/G2G4S2	0.678	0.851
G2G4S/G2G4S3	0.407	0.857
G2G4S2/G2G4S3	0.427	0.841
S1	0.991	0.991
S2	0.395	0.970
S3	0.433	0.774
S4	0.814	0.924
S	0.943	0.976
F	0.441	0.722

Stage I, sex (M/F):3/3, age (mean, range):62, 50-74;

Stage II, 0;

Stage III: sex (M/F): 6/6, age (mean, range):59.5,51-69;

Stage IV: sex (M/F):15/15, age (mean, range):58.5, 49-69.

Statistical analysis was performed by Kruskal-Wallis test (α=0.05).
M: male; F: female.

Table S7. Comparison of N-glycopeptides, N-glycopeptide ratios, and N-glycosylation features of DSHp-β with sex in BLDs or NSCLC patients.

N-glycopeptides, N-glycopeptide ratios, and N-glycosylation features	BLDs		NSCLC	
	***p* value**	**p_{adj} value**	***p* value**	**p_{adj} value**
**VVLHPN$_{241}$YSQVDIGLIK**				
G2	0.488	0.902	0.829	1.032
G2S	0.440	0.994	0.428	0.768
G2S2	0.207	0.840	0.114	0.463
G2/G2S	0.344	0.954	0.228	0.662
G2/G2S2	0.927	0.943	0.323	0.680
G2S/G2S2	0.474	0.933	0.860	0.990
**NLFLN$_{207}$HSEN$_{211}$ATAK**				
G2G2	0.972	0.972	0.710	0.963
G2G2S	0.490	0.880	0.577	0.903
G2G3	0.836	0.911	0.659	0.957
G2G2S2	0.791	0.911	0.053	0.464
G2G3S	0.096	0.734	0.887	0.967
G2G4	0.701	0.873	0.138	0.496
G2G3FS	0.791	0.928	0.231	0.639
G2G2S3	0.678	0.919	0.137	0.523
G2G3S2	0.230	0.780	0.851	1.018
G2G4S	0.064	0.645	0.891	0.954
G2G3FS2	0.167	0.847	0.579	0.883
G2G2S4	0.507	0.883	0.312	0.679
G2G3S3	0.121	0.673	0.610	0.907
G2G4S2	0.538	0.864	0.923	0.970
G2G3FS3	***0.027***	0.404	***0.025***	0.508
G2G3S4	0.483	0.921	0.280	0.682
G2G4S3	0.913	0.944	0.923	0.954
G2G2/G2G2S2	0.454	0.924	0.156	0.527
G2G2S2/G2G2S3	0.831	0.922	0.727	0.965
G2G2/G2G2S	0.517	0.875	0.859	1.008
G2G2/G2G2S3	0.704	0.859	***0.028***	0.420
G2G2/G2G2S4	0.735	0.880	0.698	0.968
G2G3/G2G3S	0.358	0.909	0.570	0.914
G2G3/G2G3S2	0.104	0.702	0.743	0.964
G2G3/G2G3S3	0.454	0.954	0.404	0.771
G2G3/G2G3S4	0.597	0.868	***0.045***	0.458
G2G3S/G2G3S2	0.846	0.905	0.067	0.508
G2G3S/G2G3S3	0.817	0.923	0.105	0.491

G2G3S/G2G3S4	0.211	0.806	0.218	0.664
G2G3S2/G2G3S3	0.700	0.890	0.298	0.673
G2G3S2/G2G3S4	0.581	0.887	0.676	0.959
G2G3S3/G2G3S4	0.584	0.868	0.821	1.043
G2G2S/G2G2S2	0.261	0.838	***0.019***	0.565
G2G2S/G2G2S3	0.658	0.912	***0.042***	0.515
G2G2S/G2G2S4	0.655	0.929	0.841	1.026
G2G2S2/G2G2S4	0.859	0.903	0.472	0.800
G2G2S3/G2G2S4	0.577	0.902	0.243	0.644
G2G3FS/G2G3FS2	0.302	0.878	0.986	0.986
G2G3FS/G2G3FS3	***0.023***	0.474	0.280	0.657
G2G3FS2/G2G3FS3	0.347	0.921	0.190	0.608
G2G4/G2G4S	0.450	0.980	0.974	0.990
G2G4/G2G4S2	0.688	0.912	0.500	0.824
G2G4/G2G4S3	0.186	0.872	0.352	0.694
G2G4S/G2G4S2	0.391	0.916	0.875	0.970
G2G4S/G2G4S3	0.112	0.685	0.086	0.476
G2G4S2/G2G4S3	0.389	0.949	0.071	0.433
G2G3FS/G2G3S	0.224	0.805	***0.012***	0.743
G2G3FS2/G2G3S2	***0.005***	0.148	0.067	0.454
G2G3FS3/G2G3S3	***<0.001***	***0.003***	0.093	0.470
S1	0.689	0.894	0.419	0.775
S2	0.080	0.694	0.462	0.805
S3	0.197	0.858	0.255	0.648
S4	0.528	0.871	0.350	0.712
S	0.296	0.903	0.871	0.984
F	0.056	0.681	0.107	0.464

A $p(p_{adj})$ value of < 0.05 was considered to be statistically significant.

For BLDs patients:

Male (n=111), age (mean, range):57.7, 37-74; female (n=115), age (mean, range):57.5, 37-73.

For NSCLC patients:

Male (n=136), age (mean, range):57.9, 37-73; female (n=127), age (mean, range):57.0, 37-73.

Table S8. Comparison of the N-glycopeptides, N-glycopeptide ratios, or N-glycosylation features of DSHp-β with age in BLDs or NSCLC patients.

N-glycopeptides, N-glycopeptide ratios, and N-glycosylation features	BLDs		NSCLC	
	p value	p_{adj} value	*p* value	p_{adj} value
**VVLHPN$_{241}$YSQVDIGLIK**				
G2	0.714	0.947	0.982	0.998
G2S	0.444	0.933	0.988	0.988
G2S2	0.113	0.861	0.654	1.050
G2/G2S	0.211	1.070	0.962	1.030
G2/G2S2	0.312	1.002	0.978	1.028
G2S/G2S2	0.471	0.927	0.332	0.920
**NLFLN$_{207}$HSEN$_{211}$ATAK**				
G2G2	0.231	0.941	***0.028***	1.721
G2G2S	***0.027***	0.811	0.670	1.022
G2G3	0.061	0.739	0.660	1.032
G2G2S2	0.085	0.868	0.980	1.014
G2G3S	0.191	1.058	0.760	1.053
G2G4	0.697	0.966	0.126	0.769
G2G3FS	0.365	1.011	0.509	1.036
G2G2S3	0.274	0.985	0.142	0.722
G2G3S2	0.431	0.973	0.790	1.048
G2G4S	0.793	0.987	0.896	1.012
G2G3FS2	0.701	0.950	0.652	1.074
G2G2S4	0.629	1.010	0.373	0.949
G2G3S3	0.386	0.980	0.851	1.039
G2G4S2	0.976	1.009	0.293	0.892
G2G3FS3	0.855	1.023	0.931	1.032
G2G3S4	0.361	1.048	0.112	0.850
G2G4S3	0.620	1.022	0.210	0.754
G2G2/G2G2S2	0.037	0.752	0.125	0.846
G2G2S2/G2G2S3	0.975	1.025	0.083	1.271
G2G2/G2G2S	***0.017***	1.021	0.090	1.093
G2G2/G2G2S3	0.524	0.969	0.204	0.889
G2G2/G2G2S4	0.655	0.999	0.229	0.777
G2G3/G2G3S	0.598	1.042	0.599	1.044
G2G3/G2G3S2	0.549	0.985	0.816	1.060
G2G3/G2G3S3	0.680	0.965	0.627	1.062
G2G3/G2G3S4	0.230	1.001	***0.042***	1.284
G2G3S/G2G3S2	0.973	1.041	0.512	1.007
G2G3S/G2G3S3	0.900	0.998	0.586	1.052
G2G3S/G2G3S4	0.997	0.997	0.207	0.841

G2G3S2/G2G3S3	0.992	1.009	0.375	0.915
G2G3S2/G2G3S4	0.916	0.998	0.097	0.982
G2G3S3/G2G3S4	0.892	1.007	0.058	1.178
G2G2S/G2G2S2	0.486	0.927	0.888	1.022
G2G2S/G2G2S3	0.666	0.991	0.128	0.712
G2G2S/G2G2S4	0.218	1.025	0.501	1.053
G2G2S2/G2G2S4	0.715	0.928	0.188	0.881
G2G2S3/G2G2S4	0.311	1.054	0.460	1.002
G2G3FS/G2G3FS2	0.811	0.989	0.459	1.037
G2G3FS/G2G3FS3	0.670	0.974	0.541	0.999
G2G3FS2/G2G3FS3	0.455	0.924	0.837	1.064
G2G4/G2G4S	0.431	0.939	0.737	1.046
G2G4/G2G4S2	0.606	1.026	0.885	1.038
G2G4/G2G4S3	0.864	1.014	0.316	0.917
G2G4S/G2G4S2	0.881	1.014	0.111	0.968
G2G4S/G2G4S3	0.648	1.014	0.781	1.059
G2G4S2/G2G4S3	0.353	1.075	0.680	1.012
G2G3FS/G2G3S	0.128	0.864	0.531	1.011
G2G3FS2/G2G3S2	0.131	0.800	0.856	1.024
G2G3FS3/G2G3S3	0.378	1.003	0.208	0.793
S1	0.421	0.987	0.248	0.795
S2	0.060	0.917	0.716	1.039
S3	0.102	0.891	0.932	1.015
S4	0.418	1.021	0.406	0.951
S	0.240	0.915	0.846	1.053
F	0.738	0.938	0.369	0.978

BLDs, n=236; NSCLC, n=272.

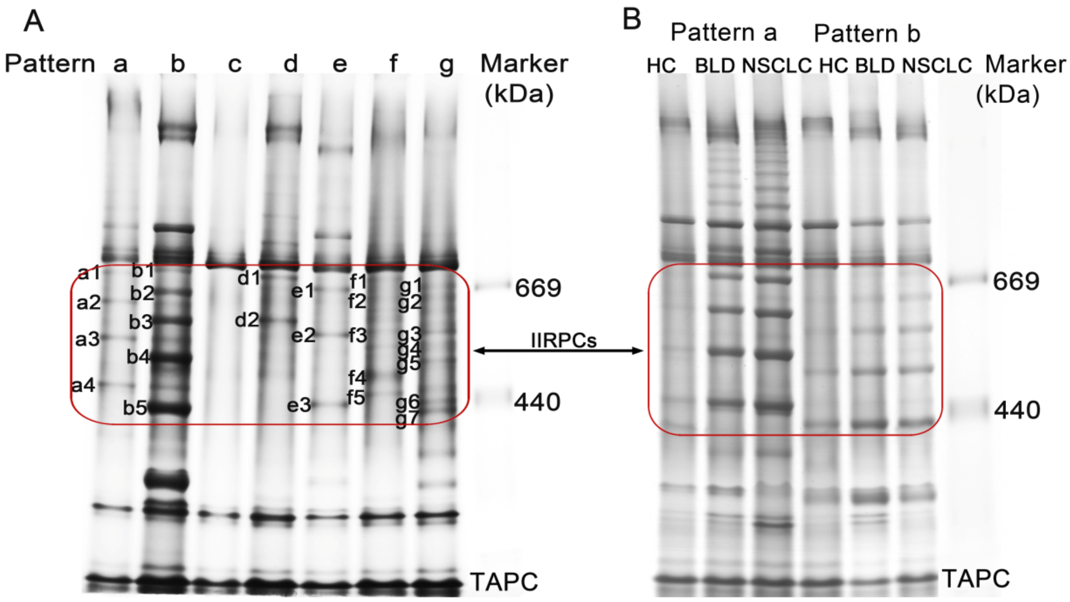

Figure S1. Serum IIRPCs isolated by native-PAGE. HC, healthy control; IIRPCs, immunoinflammation-related protein complexes; TAPC, transferrin-related protein complex.

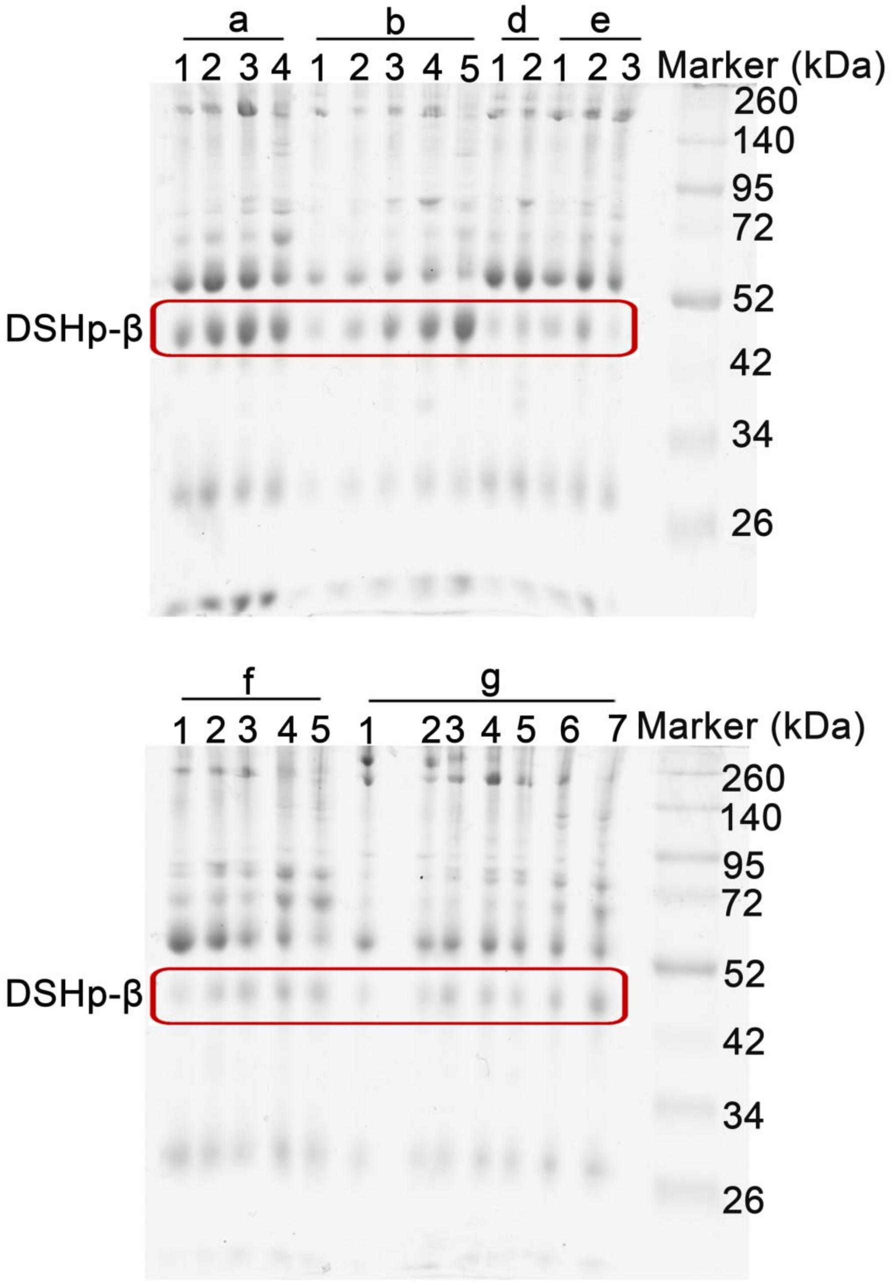

Figure S2. Isolation of DSHp-β from serum IIRPCs by SDS-PAGE. DSHp-β, disease specific Hp-β.

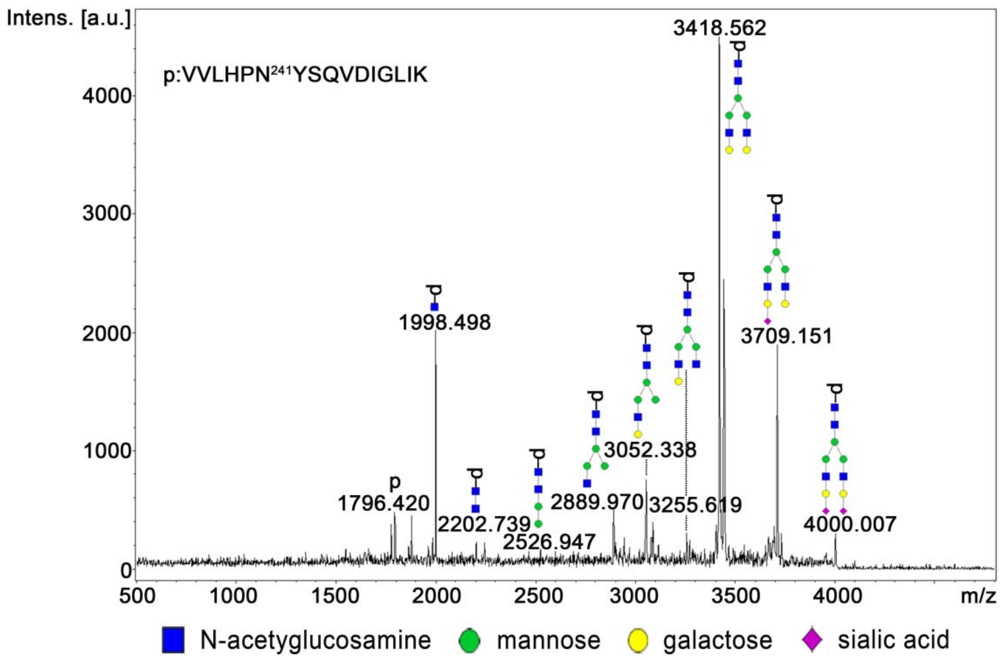

Figure S3. Tandem mass spectrum of G2S2 at *m/z* 4000.007 obtained using an UltrafleXtreme MALDI-TOF/TOF mass spectrometry.
